# A Comparative Study of Thought Fusion Beliefs and Thought Control Strategies in Patient With Obsessive-Compulsive Disorder, Major Depressive Disorder and Normal People

**Published:** 2014

**Authors:** Ahmad Amiri Pichakolaei, Samad Fahimi, Abbas Bakhshipour Roudsari, Ali Fakhari, Ebrahim Akbari, Masoumeh Rahimkhanli

**Affiliations:** 1 Department of Clinical Psychology, School of Psychology and Educational Sciences, Tabriz University, Tabriz, Iran.; 2 Associate Professor, Department of Psychology, Shool of Psychology and Educational Sciences, Tabriz University, Tabriz, Iran.; 3 Associate Professor, Department of Psychiatry, School of Medicine, Tabriz University of Medical Sciences, Tabriz, Iran.; 4 Department of Clinical Psychology, School of Psychology and Educational Sciences, Kharazmi University, Tehran, Iran

**Keywords:** Major Depression Disorder, Metacognition, Obsessive-Compulsive Disorder, Thought Control Strategies, Thought Fusion Beliefs

## Abstract

**Objective: **The present study aimed to investigate the metacognitive model of obsessive-compulsive disorder (OCD), through a comparative study of thought fusion beliefs and thought control strategies between patients with OCD, depression, and normal people.

**Methods:** This is a causal-comparative study. About 20 patients were selected with OCD, and 20 patients with major depression disorder (MDD), and 20 normal individuals. Participants completed a thought fusion instrument and thought control questionnaire. Data were analyzed using multivariate analysis of variance.

**Results: **Results indicated that patients with OCD obtained higher scores than two other groups. Also, there was a statistical significant difference between the three groups in thought control strategies and punishment, worry, and distraction subscales.

**Conclusion:** Therefore, the results of the present study supported the metacognitive model of obsessive and showed thought fusion beliefs and thought control strategies can be effective in onset and continuity of OCD.

## Introduction

Cognition theories emphasize on the prominent role of dysfunctional beliefs in recognizing causes and symptoms of continuing obsessive-compulsive disorder (OCD) (1, 2). In the new cognition theories about an anxiety disorder and specially OCD, metacognition constructs have a salient position (1, 3, 4). For example, Purdon and Clark show metacognition beliefs considered that the need for controlling the intrusive thought in creation and continue OCD is very prominent (5).

In this regard, Wells and Matthews presented a pattern for OCD and based on this pattern intrusive thought activates the metacognition beliefs which are related to the meaning of thought, and simultaneously are connected to those instrumental beliefs that are related to the behavioral responses and can decrease the evaluated danger association with obsession thoughts (6).

The metacognitive model proposes that obsession thoughts are negatively interpreted because of metacognitive beliefs about the meaning and/or dangerous consequences of having a thought. Two domains of metacognitive beliefs are implicated in the model and treatment of OCD: (a) metacognitive beliefs about the significance or importance of thoughts and feelings and (b) metacognitive beliefs about the need to perform rituals. Within the first domain, a range of different themes may be identified. For example, beliefs about intrusions can involve themes of: (a) thought-action fusion (TAF); “if I think about stabbing him, I probably will stab him”; (b) thought-event fusion (TEF); “If I think of the Devil, the Devil will appear or if I think I’ve abused her, I probably have done so”; and (c) thought-object fusion (TOF); an example is the belief that “feelings of unrest” can be transferred into books, thus contaminating them such that the feeling can never be escaped when the infected books are used (2, 3).

According to metacognition model, the activation of dysfunctional metacognition beliefs will cause negative evaluation of intrusive thought and will be a sign of threatening. This evaluation will cause extra excitements which are negative and these excitements, most of the time, are like anxiety and, as a result, that a person for decreasing his/her anxiety and controlling the cognition organization should pay attention to the ways in which they can control their thoughts (3, 6-8).

Wells and Davies found that individuals use five general strategies to control intrusive thoughts: (a) distraction (e.g., I do something that I enjoy), (b) social control (e.g., I ask my friends if they have similar thoughts), (c) worry (e.g., focus on different negative thoughts), (d) punishment (e.g., I punish myself for thinking the thought), and (e) reappraisal (e.g., I challenge the thought’s validity) (9).

Many researchers had reported a positive and significant association between the symptoms of OCD and thoughts fusion (TF) and counted it as a powerful predictor for the symptoms of OCD (3, 10, 11). For instance, in a research, Myers and Wells showed that the scores of university student in thoughts fusion inventory (TFI) have a positive and significant association with responsibility and signs of OCD. Findings showed that even when we control responsibility and worry, again the association between signs of OCD and the scores TFI would be significant (11).

A number of researches depicted that believing in the importance and reality of thoughts is not a specific OCD; rather are other disorders, especial anxiety and depression disorder, it is an obligatory fact (12-15).

For example, in the study of Abramowitz et al. the participated patients suffered from OCD, generalized anxiety disorder (GAD), panic disorder (PD), social phobia, and major depression disorder (MDD) and also there were a group of normal people. There was not any statistical significance difference in the moral TAF. About the other probabilities, there was a significance difference between OCD patients and those who suffered from social phobia, MDD, and a normal group, while they had no difference with those who suffered from panic disorder, PD and GAD (16).

About using the thought control strategy, the result of investigations showed that those who suffered from OCD in comparison with normal people had more punishment, worry, reappraisal, and social control. Those who were normal used more strategy control thought of distraction (16-18). In one case, in other researches there was a positive and significant association between depressions, rumination, and thought control strategy (19-21).

Hence, findings are paradoxical (16, 17, 19); for example, in the study of Belloch et al. they compared the thought control strategy in OCD people and MDD groups with those suffering from anxiety disorder. According to the results of this research in punishment, there was a significance association between OCD group and other groups. But in control thought strategy of worry, reappraisal, social control, and distraction, there was not any significance difference between the groups (19).

The preset study aimed to examine the metacognition model of OCD; we did this examination by comparing thought fusion beliefs and thought control strategy between the patients with OCD, MDD, and normal people.

The main hypothesis of the result is as follows:

- First: there is a difference between OCD, MDD, and normal people in thought fusion beliefs.
- Second: there is a difference between OCD, MDD groups and normal people in thought control strategy.

## Materials and Methods

***Participant and procedure***

The present study was a cross-sectional and ex-post facto investigation (causal comparative method). In this research, there were patients with OCD, MDD, and normal people of Tabriz, Iran. There were 20 patients with OCD, 20 patients with MDD, and 20 normal people. For selecting the clinical case, the method of sampling in access was used. The subjects of the research came from the clients of Bozorgmehr Psychiatric Clinic (Tabriz, Iran) who had come there for the first time and after the psychiatrist diagnosed the disorder, the cases entered into the research. In addition, 20 normal subjects were chosen among employees and university students of Tabriz University, who did not have any psychological disorder.

The criteria that allowed patients to enter into the research are as below: containing the 4^th^ edition of the Diagnostic and Statistical Manual of Mental Disorders, Fourth edition (DSM-IV), Text Revision (DSM-IV-TR) for those who suffered from OCD and MDD without contamination with disorders of axis one and two DSM-IV by diagnostic psychiatric an clinical psychologist, not receiving psychological therapy or medical treatments before entering into the research, age range 18-50, and having at least with high school graduation level.

The exclusive criteria for research sampling patients are as below: having contamination with disorder with axis one and two, having psychotic disorder and addiction, and containing complete criterion of personality in axis two.

The criteria of entrance for normal people are as below: lack of history of psychological disorder and without a diagnosis of psychological disorder in axis one and two by interview of the clinical psychologist with using structured clinical interview for DSM-IV.

***Measure***

*The TFI (Wells et al., 2001)*

This is a 14 item self-report measure assessing metacognitive beliefs about the meaning and power of thoughts. It was designed to measure the three types of thought fusion implicated in the metacognitive model: TAF (e.g., ‘‘if I have thoughts about harming someone I will act on them”), TEF (e.g., “my thoughts alone have the power to change the course of events”) and TOF (e.g., “my feelings can be transferred into objects.”) (22). Gwilliam et al. reported good internal consistency with a Cronbach’s alpha 0.89 for the scale. Corrected item-total correlations ranged from 0.35 to 0.78. The TFI significantly correlated with measures of related concepts (the Metacognition Questionnare-30 [MCQ-30; Wells and Cartwright-Hatton] and the TAF Scale [Shafran et al.]) (23-25). The amount of variance shared with these questionnaires (30.25% and 20.25%, respectively) suggests the TFI measures a related but distinct construct. Test-retest reliability over 3 months was acceptable with a coefficient of 0.69 (26). Khoramdel et al. in their final investigation about internal consistency found the alpha coefficients for the general factor the index was 0.87 and for the first, second, third factors, and split-half coefficient, it was 0.77, 0.82, 0.80, and 0.73, respectively. The unit index of convergent inputs between the test of mixed thought and questionnaire of mixed thought was 0.65 (27).

*Thought control questionnaire* *(Wells and Davies, 1994)*

This 30-item self-report instrument assesses the frequency of using different strategies to control negative unwanted thoughts. The instrument includes five empirically derived subscales: distraction, punishment, reappraisal, social control, and worry. The items are scored on a 4-point Likert scale from 1 = “never” to 4 = “almost always."”. The original five factor structure of the thought control questionnaire (TCQ) has been replicated using exploratory factor analysis in both non-clinical (9) and clinical samples (20). However, its reliability has been questioned by Fehm and Hoyer (20, 28) who also pointed out the existence of items with problematic factor loadings, and recommended item refinement of the TCQ. In a confirmatory factor analysis (29), the five-factor structure of the TCQ was confirmed, but the length of the instrument was dramatically reduced in order to preserve good psychometric properties. A new 16-item TCQ version called reduced TCQ (TCQ-r), was postulated in order to improve the interpretation of the factor contents. Consistent with previous findings, only the subscales of punishment and worry were shown to be related, in non-clinical subjects, to symptom measures of depression, OCD and worry (29). The number of items on each TCQ-r subscale and their respective Cronbach’s alphas were as follows: distraction: four items, α = 0.74; social control: four items, α = 0.70; worry: two items, α = 0.42; punishment: three items, α = 0.81; and re-appraisal: three items, α = 0.72. The reliability of the overall scale was α = 0.75. In the present article, we used this TCQ-r. In a research done in Iran between 100 people, the alpha coefficients for the whole questionnaire was 0.81 and for punishment it was 0.76, for a reappraisal it was 0.70, for worry it was 0.70, and the social control was reported (30). In the study of Fata et al., with Iranian sample using exploratory factor analysis, five factors could be interpreted as a distraction, worry, social control, punishment, and reappraisal for thought control questionnaire identified. Besides, the internal consistency of the scale factor controls was 0.64-0.74, respectively (31).

***Overview of data analysis***

The pattern of the present study according to the topic was the aim and hypothesis of this research and descriptive and ex-post facto. Statistical Package for Social Sciences (SPSS) for Windows 17.0 (SPSS Inc., Chicago, IL, USA) was used for classifying, processing, and analyzing the inputs. For determining the difference between the variables, multivariate analysis of variance (MANOVA) method was used.

## Results

Descriptive finding for each groups of OCD, MDD and normal people are shown in table 1.

Descriptive finding for each group of OCD, MDD and normal people are shown in table 1.

In table 2, descriptive findings mean and standard deviation, thought fusion and subscales and thought control strategy and subscales of each group of OCD, MDD, and normal people are all represented.

MANOVA was used for investigating the hypothesis of the research. Related information about MANOVA is presented in table 3.

**Table 1:** Descriptive finding for each groups of obsessive compulsive disorder (OCD), major depression disorder (MDD) and normal people

**Variable**	**Group**	**Obsessive-compulsive disorder**	**Major depression disorder**	**Normal patients**
**Sex**	Male	09	05	13
	Female	11	15	07
**Education**	High school graduate	07	14	10
	BSc†	08	05	08
	MSc‡	05	01	02
**Age**	Mean (standard deviation)	35.26 (5.08)	33.80 (0.73)	22.90 (2.74)

† Bachelor of science;

‡ Master of science

**Table 2:** Mean and standard deviation (SD) for thought fusion and subscales and thought control strategy and their subscales

**Group** **Variables**	**Obsessive-compulsive disorder**		**Major depression disorder**		**Normal people**	
	**Mean**	**SD** †	**Mean**	**SD** †	**Mean**	**SD** †
**Thought fusion**	78.70	19.72	54.05	18.60	49.85	22.01
**Thought-action fusion**	18.95	09.12	12.85	07.01	10.75	06.67
**Thought event action**	34.05	08.09	26.05	08.38	22.10	10.68
**Thought-object fusion**	25.70	08.37	15.15	07.54	17.01	09.54
**Thought control**	69.65	08.48	70.40	09.72	66.90	09.31
**Punishment**	14.70	02.77	13.50	02.78	10.55	02.91
**Worry**	11.65	03.49	15.12	04.32	09.70	02.10
**Reappraisal**	15.15	02.83	16.80	02.36	15.50	03.28
**Social control**	13.65	03.40	12.30	03.27	17.10	03.40
**Distraction**	14.50	02.92	15.50	03.11	13.45	02.05

† Standard deviation

**Table 3:** Results of multivariate analysis to compare variables of thought fusion and thought control

**Variable**	**Sum of squares**	**df** †	**Mean of square**	**F**	**Significant**
**Thought fusion**	9717.23	2	4858.61	11.95	0.0001
**Thought-action fusion**	725.73	2	0362.61	06.15	0.0040
**Thought event action**	1482.70	2	0741.35	07.86	0.0010
**Thought-object fusion**	1269.43	2	0634.71	08.72	0.0001
**Thought control**	0135.82	2	0067.91	00.80	0.4500
**Punishment**	0182.43	2	0091.21	10.83	0.0000
**Worry**	0302.23	2	0010.04	10.50	0.2310
**Reappraisal**	0095.43	2	0047.71	41.04	0.0230
**Social control**	0245.10	2	0122.55	10.83	0.0000
**Distraction**	0026.43	2	0013.22	01.61	0.2080

† Degree of freedom

**Table 4:** Least significant difference for comparison of study groups in the variables thought fusion and thought control and its components

**Comparative variables**	**1 group**	**2 group**	**Mean Difference**	**Standard error**	**Significant**
**Thought fusion**	OCD†	MDD‡	24.65	06.37	0.0001
		Normal	28.85	63.37	0.0001
**Thought-action fusion**	OCD†	MDD‡	06.10	02.42	0.0150
		Normal	08.20	02.42	0.0010
**Thought event action**	OCD†	MDD‡	8	03.06	0.0120
		Normal	11.95	03.06	0.0001
**Thought-object fusion**	OCD†	MDD‡	10.55	02.69	0.0001
		Normal	08.70	12.69	0.0020
**Punishment**	OCD†	Normal	04.15	00.90	0.0001
	MDD‡	Normal	02.95	00.90	0.0020
**Worry**	MDD‡	Normal	03.05	01.08	0.0070
**Distraction**	Normal	OCD†	03.45	01.06	0.0020
		MDD‡	04.80	01.06	0.0001

† Obsessive compulsive disorder;

‡ Major depression disorder

The proportions of F which are made in 0.001 are all significant difference by Wilks’ Lambda test (df = 20; F = 4.70) (p = 0.001). Therefore, the general hypothesis of the research about the difference of groups in investigated variables is certified.

According to the represented results which are in table 3, it was pointed out that there was a significance difference between these three groups of subjects in variables of thought fusion and subscales TAF, TEF, TOF, and there was also a difference in variable of strategy control thought subscales, punishment, worry, and distraction. However, in variables reappraisal, and social control, in these three groups, no sign of significance difference had been found. Given to the significance difference of the variable, for pointing out the clear differences between these three groups, the test of least significant difference (LSD) was used. The results of this test are represented in table 4.

The results of table 4 show that there was a statistical significance difference between those suffering from OCD, MDD, and normal people in variable of thought fusion. It shows that those who suffer from OCD have more thought fusion compared with two other groups. Furthermore, suffering from OCD in variable of TAF, TEF, and TOF have higher scores in compare with two other groups. According to table 4, there was a statistical significance difference between those suffering from OCD and normal people in variable of punishment; it means that this group uses more thought control strategy punishment in compare whit normal people. In this very variable, suffering from MDD use thought control strategy punishment in compared to normal people. Another variable in this research is worry that there was a difference between those suffering from MDD and normal people. It means that MDD people use more thought control strategy i.e. worry, in comparing with normal people. Moreover, the variable of thought control strategy of distraction, between those groups was different. It shows that normal subjects are confronted with the intrusive thought; they use thought control strategy distraction than the two other groups.

## Discussion

The aim of the present research was to examine the metacognition model of OCD; we did this examination by comparing thought fusion beliefs and thought control strategy between those who patients OCD, MDD, and normal people.

The results of this research confirmed meaningful statistically significant difference in average scores of mixed thought in the three groups of patients. The results of this research, with the help from MANOVA test, pointed out that there was a statistical significant difference between the average scores of the groups. For the difference test among groups, the test of LSD was used which showed that there was a statistical significant difference in average scores of thought fusion and its subscales between OCD group with MDD and normal people. It means that there was a statistical significant difference in average scores of TAF, TEF, and TOF between OCD group and the two groups of MDD and normal people. These findings are the same with the findings of previous researches (10, 11, 23, 32).

In “Wells” opinion, intrusive thought causes the activation of metacognitive beliefs (thought fusion) about the meaning of thought. These beliefs are about importance, meaning, and power of thought. The beliefs about power and meaning of thought and emotions in the metacognitive theory points out the thought fusion that in this aspect, the border between thought and action, thought and event, and thought and object will be removed. The activation of these useless metacognitive beliefs by the intrusive thought causes negative evaluation of thought and emerges as a sign of threat. This evaluation causes extra negative excitement that most of the times emerge as anxiety (2, 3).

Data from path analyses and structural equation modeling work on obsessive-compulsive symptoms has shown that the metacognitive model is depicting the association between thought fusion beliefs, appraisal, and beliefs about rituals and symptoms fit the data well in non-patients. Tests of alternative rival models of relationships among these variables did not fit the data (32).

Several researchers in the field of obsessive-compulsive symptom shave demonstrated specific contributions of metacognitive beliefs to symptoms over and above the contribution made by another non-metacognitive belief domain (10, 23, 32). Gwilliam et al. examined whether metacognitive beliefs or responsibility-related cognitions predicted obsessive-compulsive symptoms in non-patients. The results indicate both the responsibility and the fusion-related belief domains were positively correlated with symptoms. However, the thought fusion beliefs were the strongest correlates and the relationship between responsibility and symptoms were no longer present when metacognitions were accounted for (23).

Many researchers have found a positive correlation between TAF and signs of depression and have showed that there was not any statistical significant difference between the scores of OCD and MDD (16, 25, 33).

However, in the present research, there has been a statistical significant difference between these two groups which are not in consistent with previous researches. To explain these finding, it must be mention that in previous researches, TAF consisted of two types of TAF: moral and probability TAF. And there was not any statistical significant difference in average scores of moral TAF between OCD and MDD groups, but there was a statistical significant difference in average scores of probable TAF between the groups (16, 25, 33). Therefore, that is why the present research is not in consistent with the previous researches. 

Moreover, the results of this research confirm the second hypothesis of research, that is, the average scores of thought control and its small scales between OCD, MDD groups and normal people are different. The result of the research, with the use of MANOVA test, showed that there was a statistical significant difference between average scores of punishment, worry, and distraction strategies. These findings are in consistence with other researches (16-19).

Test of difference of paired average scores between the groups, with the use of LSD test, showed that OCD and MDD groups use the thought control strategy of punishment more than normal people. One of the other findings of the research was the statistical significant difference in average thought control strategy of worry between MDD group and normal people. Furthermore, there was a statistical significant difference between average scores of thought strategy of distraction in normal people in comparing with OCD and MDD groups. It means that normal people make use of thought control strategies of distraction for controlling intrusive and unwanted thought in comparing with OCD and MDD groups. These findings are also in consistence with previous researches. Besides, there was not any statistical significant difference between average scores of thought control strategy of worry in OCD and two other groups, and it is in not consistence with previous findings (16-18).

The study showed that when we try to control intrusive and unwanted thoughts, such as obsession thoughts, it causes reversing, and these suppressed thoughts will relapse and, as a result, this thought will be strengthened (34-38).

Therefore, it seems that those who suffer from anxiety disorder such as OCD try later to control such thoughts, and it would increase the abundant of thought. And they would be ready to strengthen these disorders.

There is a large literature on the effects of thought suppression, but it has produced equivocal results in terms of the reliability of immediate or delayed effects of trying to suppress a target thought. However, the overriding conclusion is that trying to suppress a thought is not entirely effective. This generally supports the idea that metacognitive thought control strategies aimed at removing thoughts from consciousness are likely to be inefficient, yet this is a strategy often reported by patients (3). For example, in the research of Purdon et al. they asked OCD patients to suppress their obsession thoughts and to find showed that there were paradoxical results (38).

Wells pointed out three mechanisms that could explain the escalation of barriers for thinking: first, attempts to suppress thoughts may cause an enhanced awareness of unwanted thoughts. Second, attempts to ruminate on intrusions or mentally neutralize them can maintain preoccupation with mental events, making intrusion more likely. Third, activities such as reiterated checking or cleaning establish relations between the domain of stimuli and obtrusion, such that a widening array of stimuli! Actions can start obtrusion (2).

The general result of the present research supports the metacognitive model of OCD. It means they support the beliefs about unwanted thoughts and said that these thoughts cause negative prediction that this process wants some strategies for control and neutralize of the unwanted thoughts, and this action will prone an individual into a person with OCD and enhanced the disorder symptoms.

## Authors' contributions

The first, second and third author conceived and designed the evaluation. The first, second and fourth author collected the clinical data. The first and second author interpreted the clinical data and performed parts of the statistical analysis and drafted the manuscript. The third, fourth, fifth and sixth author revised the manuscript and performed the statistical analysis and revised the manuscript. All authors read and approved the final manuscript.

## Notes

**Declaration of interest:** None.
